# Functional Brain Network of Trait Impulsivity: Whole‐Brain Functional Connectivity Predicts Self‐Reported Impulsivity

**DOI:** 10.1002/hbm.70059

**Published:** 2024-10-29

**Authors:** Philippa Hüpen, Himanshu Kumar, Dario Müller, Ramakrishnan Swaminathan, Ute Habel, Carmen Weidler

**Affiliations:** ^1^ Department of Psychiatry, Psychotherapy and Psychosomatics, Faculty of Medicine RWTH Aachen Aachen Germany; ^2^ JARA ‐ Translational Brain Medicine Aachen Germany; ^3^ Department of Applied Mechanics and Biomedical Engineering Indian Institute of Technology Madras Chennai India; ^4^ Institute of Neuroscience and Medicine: JARA‐Institute Brain Structure Function Relationship (INM 10) Research Center Jülich Jülich Germany

**Keywords:** impulsivity, individual differences, machine learning, resting‐state functional connectivity

## Abstract

Given impulsivity's multidimensional nature and its implications across various aspects of human behavior, a comprehensive understanding of functional brain circuits associated with this trait is warranted. In the current study, we utilized whole‐brain resting‐state functional connectivity data of healthy males (*n* = 156) to identify a network of connections predictive of an individual's impulsivity, as assessed by the Barratt Impulsiveness Scale (BIS)‐11. Our participants were selected, in part, based on their self‐reported BIS‐11 impulsivity scores. Specifically, individuals who reported high or low trait impulsivity scores during screening were selected first, followed by those with intermediate impulsivity levels. This enabled us to include participants with rare, extreme scores and to cover the entire BIS‐11 impulsivity spectrum. We employed repeated K‐fold cross‐validation for feature‐selection and used stratified 10‐fold cross‐validation to train and test our models. Our findings revealed a widespread neural network associated with trait impulsivity and a notable correlation between predicted and observed scores. Feature importance and node degree were assessed to highlight specific nodes and edges within the impulsivity network, revealing previously overlooked key brain regions, such as the cerebellum, brainstem, and temporal lobe, while supporting previous findings on the basal ganglia‐thalamo‐prefrontal network and the prefrontal‐motor strip network in relation to impulsiveness. This deepened understanding establishes a foundation for identifying alterations in functional brain networks associated with dysfunctional impulsivity.

## Graphical abstract

This study utilized machine learning to analyze whole‐brain functional connectivity data, mapping trait impulsivity. Findings revealed previously overlooked key brain regions within the impulsivity network, such as the cerebellum, brainstem, and temporal lobe, while supporting previous research on the basal ganglia‐thalamo‐prefrontal and the prefrontal‐motor strip network.

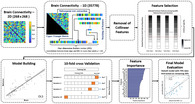

Impulsivity is a multidimensional personality trait characterized by the tendency to make rapid, premature actions without adequate foresight (Dalley and Robbins 2017; International Society for Research on Impulsivity 2021). It exists along a spectrum in both healthy individuals and various psychiatric populations, aligning with recent psychiatric classification systems emphasizing dimensional symptom definitions transcending traditional categories and reflecting extremes of normative predispositions (Cuthbert and Insel 2013; Dalley and Robbins 2017). Dysfunctional impulsivity is prevalent in many psychiatric disorders, such as Substance Use Disorder, Attention‐Deficit/Hyperactivity Disorder (ADHD), Borderline Personality Disorder, or Intermittent Explosive Disorder. Gaining a thorough neurobiological understanding of impulsivity offers potential insights into the shared and unique etiologic risk factors for psychopathology. Most studies that aim to investigate neural substrates related to impulsivity focus on psychiatric patients. These investigations consistently highlight the role of cortico‐striatal systems in impulsiveness (Dalley and Robbins 2017; Mitchell and Potenza 2014). Yet, the investigation of pathological samples is only one aspect of studying multidimensional psychological traits. To fully comprehend factors influencing the development and expression of such traits, it is essential to explore their neural substrates dimensionally in healthy individuals as well. Drawing insights from patient studies, region‐of‐interest studies using basal ganglia seeds investigated associations between resting‐state functional connectivity and impulsivity (Angelides, Gupta, and Vickery 2017; Korponay et al. 2017). The results highlight the involvement of striatal‐cortical and basal ganglia‐thalamo‐cortical circuitry in impulsivity, also in healthy individuals.

A data‐driven study using graph theory analyses examined healthy individuals' brain network organization across the impulsivity spectrum. Results revealed distinct modular organizations of whole‐brain networks in high as compared to low impulsive individuals. Specifically, results indicated increased network segregation between regulatory prefrontal and subcortical structures in individuals with high levels of impulsivity, whereas in less impulsive individuals, these networks were more integrated (Davis et al. 2013). These results again suggest that the basal ganglia‐thalamo‐cortical brain circuits play a crucial role in underpinning impulsiveness.

Furthermore, the cerebellum has recently come to the fore as a region associated with impulsivity. Specifically, it has been proposed that the cerebellum may serve as a modulatory region, involved in different forms of impulsivity (Angelides, Gupta, and Vickery 2017), by interacting with the prefrontal cortex (Miquel et al. 2019). A data‐driven analysis of the cerebro‐cerebellar system has supported this initial working hypothesis, suggesting its role in several aspects of impulsiveness (Abdallah et al. 2020).

In addition to the functional results, structural brain alterations have been reported as a function of impulsivity. Particularly, volume and cortical thickness of brain regions known to be implicated in reward‐related decision‐making (e.g., orbitofrontal cortex, anterior cingulate cortex) show associations with impulsivity (Cho et al. 2013; Matsuo et al. 2009; Schilling et al. 2012). However, the reported direction of effects often diverges.

While previous studies have explored brain correlates of impulsivity, their limited sample sizes may have contributed to the heterogeneous findings on brain correlates. Furthermore, some studies only performed region‐of‐interest analyses. In the current study, we aim to complement previous findings on functional brain‐networks underlying impulsivity by identifying the most relevant features from whole‐brain resting‐state functional connectivity in a sample of healthy individuals covering the entire dimension of impulsivity as assessed by a self‐report instrument, the BIS‐11. Another shortcoming of previous studies investigating neural correlates of trait impulsivity (assessed through self‐report questionnaires) is the random selection of participants, which often results in missing individuals with very extreme high or low impulsivity scores. In contrast, our study specifically preselected participants based on their self‐reported impulsivity scores to address this issue. Before study participation, individuals completed the BIS‐11 questionnaire, and those scoring at the extremes were invited to participate in the actual studies. Subsequently, individuals with intermediate BIS‐11 scores were also invited (see Method section). This approach makes it possible to investigate even the rare extremes, thereby enabling a comprehensive mapping of the entire trait impulsivity spectrum.

We employed a data‐driven whole‐brain approach to examine whether self‐reported impulsivity can be predicted from an individual's pattern of functional resting‐state connectivity without any a priori selection of networks in a sample of healthy male individuals. We used cross‐validation predictions, leading to a more conservative inference about brain‐behavior relationships, more robust results, and protection against overfitting. Furthermore, fMRI connectivity generates complex, high‐dimensional data, posing challenges for traditional analysis methods, whereas machine learning techniques excel in navigating this complexity, enabling the discovery of subtle patterns and relationships that might otherwise remain overlooked.

To identify the most important networks for impulsivity, we assessed the importance of functional connections (feature importance) and the most connected nodes (node degree). These measures provide valuable information on network properties and help identify potential clinically relevant network hubs.

## 1 Materials and Methods

### 1.1 Experimental Design

Data used in the current study was pooled from three studies that investigate neural correlates of impulsive behavior within the framework of the International Research Training Group 2150 on the Neuroscience of Modulating Aggression and Impulsivity in Psychopathology. All procedures were in accordance with the Declaration of Helsinki and all three individual studies were approved by the University's Ethics Committee (Ethics Committee at the RWTH Aachen Faculty of Medicine).

### 1.2 Participants

All participants met the following inclusion criteria: male gender, aged between 18 and 50 years, right‐handedness, high proficiency in the German language, no current substance dependency, no neurological diseases, no head injury. Participants were excluded in case of any MRI contraindications and if they had any MRI contraindications or a history of psychiatric disorders, as determined by the Structured Clinical Interview for DSM‐IV or DSM‐5.

**FIGURE 1:**
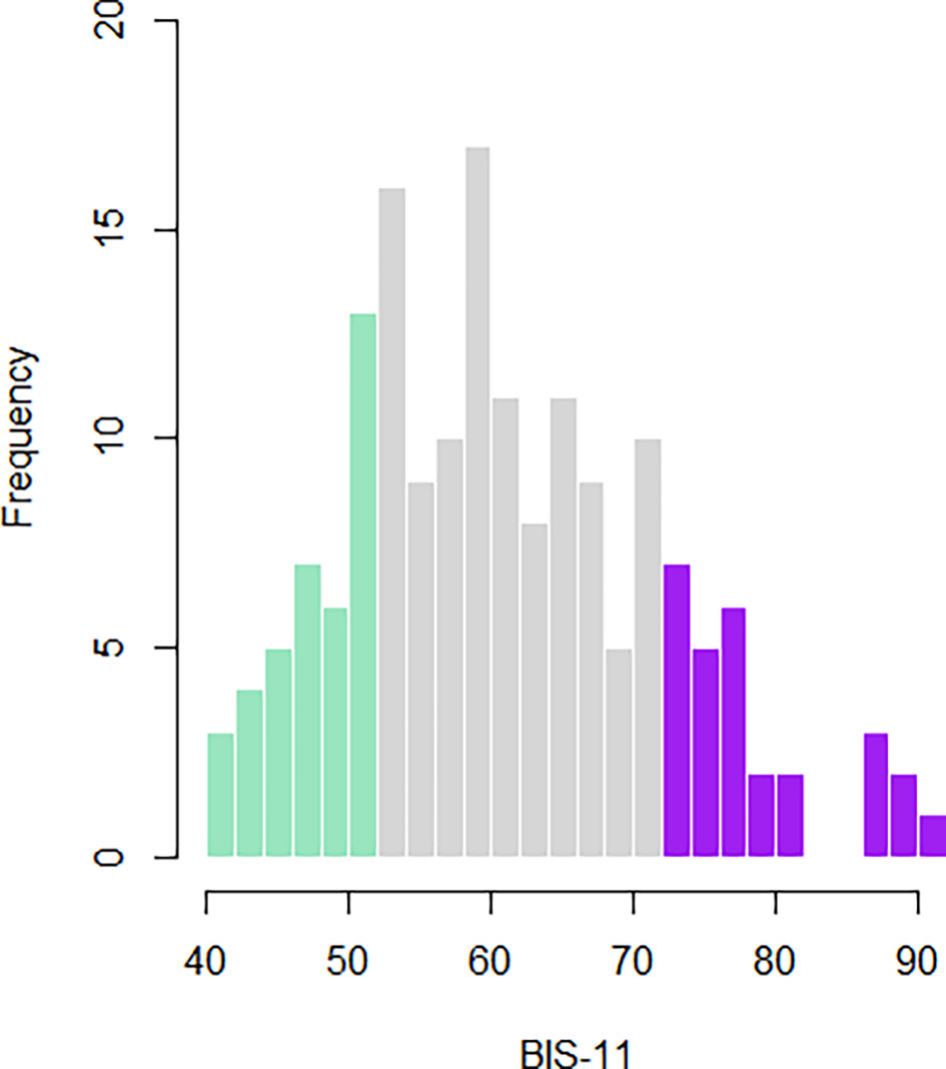
Distribution of BIS‐11 scores in the present sample.

In total, individual resting‐state data sets from a total of 156 healthy male participants were included in the current study. Their age ranged from 18 to 44 years (*M* = 24.28, *SD* = 5.17). Self‐reported BIS‐11 impulsivity scores ranged from 40 to 92 (*M* = 61.31, *SD* = 10.98). Figure 1 depicts the distribution of impulsivity scores in our sample.

Participants were selected, in part, based on their self‐reported impulsivity levels, as assessed by the German version of the Barratt Impulsiveness Scale (BIS)‐11 (Patton, Stanford, and Barratt 1995; Stanford et al. 2009). The BIS‐11 is one of the most widely used self‐ report instruments for assessing trait impulsiveness and comprises 30 items to be rated on a 4‐point Likert scale reflecting the frequency of occurrence. In this work, we took the sum of all items as the total score (after reversing scores for appropriate items), where higher total scores reflect greater levels of trait impulsiveness. Total BIS‐11 scores of 72 or above have been mentioned to reflect high impulsiveness and scores below 52 to classify low impulsiveness/over‐controlled behavior. Scores between 52 and 71 are thought of as within normal limits for impulsiveness (Stanford et al. 2009). Specifically, two of the three studies from which the current data were pooled explicitly screened for high and low levels of trait impulsiveness as assessed by the BIS‐11. Total BIS‐11 scores of 72 or above were used to classify high impulsiveness and scores below 52 were used to classify low impulsiveness/over‐controlled behavior, as suggested by previous literature (Stanford et al. 2009). In these two studies, individuals who reported high or low trait impulsivity scores during screening were selected first, followed by those with intermediate impulsivity levels. This enabled us to cover the entire BIS‐11 impulsivity spectrum. As there were no specific requirements for BIS‐11 scores in the third study, participants completed the BIS‐11 upon inclusion in the study.

Importantly, as we aim to investigate impulsivity in a dimensional fashion, we did not create groups according to impulsivity scores but used regression‐based analyses.

Resting‐state data of one of the studies was previously analyzed and has led to two publications (Müller et al. 2023; Weidler et al. 2024) not related to the research question of the current manuscript. The total number of overlapping participants included in this analysis and the previously published results is 34 participants. The remaining resting‐state data used in this current study has not been analyzed and/or published elsewhere.

### 1.3 Image Acquisition

Measurements for all three studies included in this manuscript were performed on the same MRI scanner, a Siemens 3T MAGNETOM Prisma Fit system with a 20‐channel head coil (Siemens AG; Erlangen, Germany). Functional MRI was performed using a spin‐echo EPI sequence in 34 slices (interleaved sequence, TR = 2000 ms, TE = 28 ms, flip angle = 77°, voxel size = 3 × 3 × 3 mm^3^, FOV = 192 × 192 mm^2^, slice thickness = 3.0 mm, matrix = 64 × 64).

Structural images were collected using a T1‐weighted MPRAGE sequence covering the whole brain. Parameters were slightly different for participants in study two (176 slices, TR = 2000 ms, TE = 3.03 ms, flip angle = 9°, voxel size = 1 × 1 × 1 mm^3^, FOV = 256 × 256 mm^2^, slice thickness = 1.00 mm) than for participants in study one and three (176 slices, TR = 2300 ms, TE = 2.98 ms, flip angle = 9°, voxel size = 1 × 1 × 1 mm^3^, FOV = 256 × 256 mm^2^, slice thickness = 1.00 mm).

The entire resting‐state measurement lasted 8 min, resulting in a total of 240 volumes. All participants received the instruction to focus on a fixation cross presented during the resting‐state measurement and let their thoughts wander freely.

### 1.4 Preprocessing

Functional and anatomical data were preprocessed through the CONN toolbox (Whitfield‐Gabrieli and Nieto‐Castanon 2012), release 22.a (Nieto‐Castanon and Whitfield‐Gabrieli 2021) implemented in SPM (Penny et al. 2007), release 12.7771, using a flexible preprocessing pipeline (Nieto‐Castanon 2020b) including realignment with correction of susceptibility distortion interactions, outlier detection, direct segmentation and MNI‐space normalization, and smoothing. Functional data were realigned using SPM realign & unwarp procedure (Andersson et al. 2001), where all scans were coregistered to a reference image (first scan of the first session) using a least squares approach and a six parameter (rigid body) transformation (Friston et al. 1995) and resampled using b‐spline interpolation to correct for motion and magnetic susceptibility interactions. Potential outlier scans were automatically identified using ART (Whitfield‐Gabrieli, Nieto‐Castanon, and Ghosh 2011) as acquisitions with framewise displacement above 0.9 mm or global BOLD signal changes above five standard deviations (Nieto‐Castanon 2022; Power et al. 2014), and a reference BOLD image was computed for each subject by averaging all scans excluding outliers. Functional and anatomical data were normalized into standard MNI space, segmented into grey matter, white matter, and CSF tissue classes, and resampled to 3 mm isotropic voxels following a direct normalization procedure (Calhoun et al. 2017; Nieto‐Castanon 2022) using SPM unified segmentation and normalization algorithm (Ashburner 2007; Ashburner and Friston 2005) with the default IXI‐549 tissue probability map template. Finally, functional data were smoothed using spatial convolution with a Gaussian kernel of 8 mm full‐width‐half‐maximum (FWHM).

### 1.5 Denoising

In addition, functional data were denoised using a standard denoising pipeline (Nieto‐Castanon 2020a) including the regression of potential confounding effects characterized by white matter timeseries (5 CompCor noise components), CSF timeseries (5 CompCor noise components), motion parameters and their first order derivatives (12 factors) (Friston et al. 1996), outlier scans (33 factors) (Whitfield‐Gabrieli, Nieto‐Castanon, and Ghosh 2011), session effects and their first order derivatives (2 factors), and linear trends (2 factors) within each functional run, followed by bandpass frequency filtering of the BOLD timeseries (Hallquist, Hwang, and Luna 2013) between 0.008 and 0.09 Hz. CompCor (Behzadi et al. 2007; Chai et al. 2012) noise components within white matter and CSF were estimated by computing the average BOLD signal as well as the largest principal components orthogonal to the BOLD average, motion parameters, and outlier scans within each subject's eroded segmentation masks.

Importantly, given that head motion confounds analyses of functional connectivity, we confirmed that BIS‐11 scores were not correlated with average framewise displacement during resting‐state fMRI (*r* = 0.08, *p* = 0.32).

### 1.6 Statistical Analysis

#### 1.6.1 First‐Level Analysis

Individual ROI‐to‐ROI connectivity matrices were calculated using the Fisher‐transformed bivariate correlation coefficient between each pair of ROIs. ROIs were defined using the Shen functional brain atlas which has high parcellation accuracy and spatial coherence, defining 268 regions (Shen et al. 2013). Individual ROI‐to‐ROI matrices were extracted from CONN for further analysis.

### 1.7 Functional Network Construction

**FIGURE 2:**
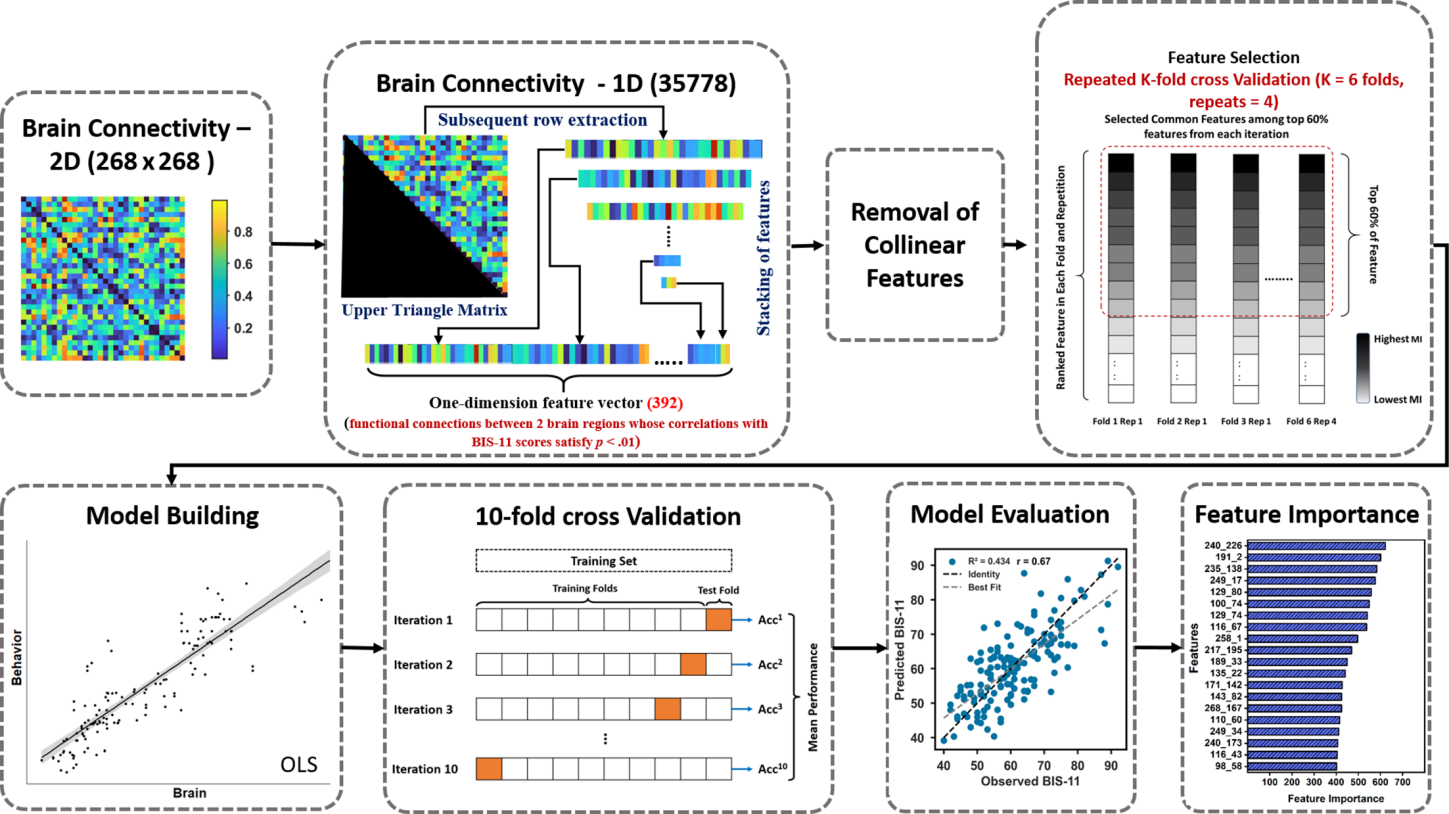
Proposed pipeline for construction of trait impulsivity brain network and prediction of individual BIS‐11 scores using whole‐brain functional connectivity data.

The resulting 268 × 268 correlation matrices represented the set of connections or edges in each individual's connectivity profile. These resting‐state whole‐brain connectivity profiles were used to predict individuals' impulsivity scores. Our pipeline was loosely based on the connectome‐based predictive modeling approach by (Shen et al. 2017). Briefly, our pipeline comprised four comprehensive steps: feature selection, model building, and feature importance assessment (see Figure 2 for the workflow of our pipeline). We first examined which connections/edges showed significant (*p* < 0.01) correlations with impulsivity scores for each subject. The negative and positive correlations were considered together as a single feature vector for each subject by concatenating them and then using the combined vector as input for the subsequent analysis. Thus, in our work features represent functional connections between two brain regions. The model was built using ordinary least squares regression and evaluated using several different metrics. Finally, we examined feature importance using *t‐*statistics.

### 1.8 Feature Reduction and Selection

Feature selection was performed to reduce the dimensionality and noise of the input features. Three steps have been employed for feature selection:

1. Removing collinear features: To identify collinear features, we examined their correlation coefficients. Features with a correlation coefficient of ≥ 0.8 were considered collinear. Among pairs of collinear features, those with the highest mutual information (MI) criterion were retained. MI is defined as the measure of the amount of information obtained about one variable through another variable. Therefore, we kept the features with the highest MI among the collinear ones and removed the others. This implies that when dealing with collinear features in the dataset, we selected the ones that provide the most unique information for further analysis. Once collinear features were identified, we retained the subset of non‐collinear features in the dataset. For two discrete random variables *X* and *Y*, MI is defined as
$$I\left(X;Y\right)=\sum_{x,y}P_{\mathrm{XY}}\left(x,y\right)\log\frac{P_{\mathrm{XY}}\left(x,y\right)}{P_{X}\left(x\right)P_{Y}\left(y\right)}$$
  where *P*_*XY*_(*x,y*) is the joint probability distribution of *X* and *Y*, and *P*_*X*_(*x*) and *P*_*Y*_(*y*) are the marginal probability distributions of *X* and *Y*.
2. We then conducted linear regression with the remaining features using repeated K‐fold cross‐validation (with *K* = 6 folds and repeats = 4). Specifically, in K‐fold cross‐validation, the dataset is divided into K subsets (folds, i.e., six in our case), with the model trained on K‐1 (five) folds and tested on the remaining fold. This process is done for K times, each time with a different fold as the test set. To further reduce the variance caused by random data splitting, the entire K‐fold cross‐validation process is repeated multiple times (i.e., four repeats in our case), each time with different random splits (see Figure 2, “Feature Selection”). Performance metrics are then averaged across the K folds to obtain an overall estimate. This approach ensures a more reliable estimate of the model's performance, reducing variance arising from the randomness in data splitting. Consequently, the acquired feature rankings were performed 24 (6 × 4) times. This method allowed us to estimate the MI between each feature and the BIS, and ranking them in each repetition.
3. Finally, the Common Features That Were Consistently Ranked in the Top 60% Across All Repeats Were Selected.

### 1.9 Model Building—Linear Regression

**FIGURE 3:**
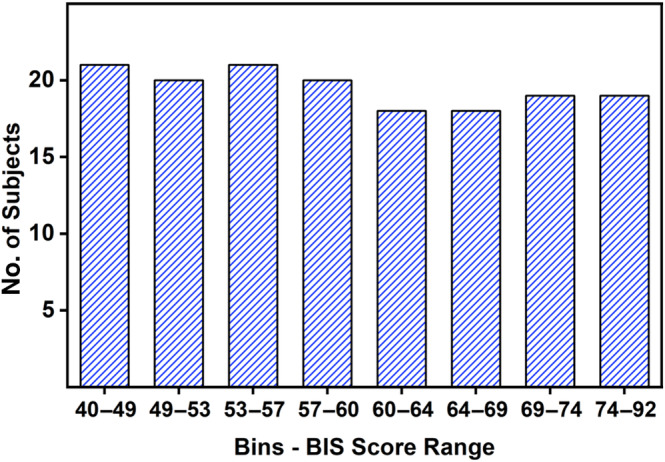
Quantile Binning of BIS‐11 scores for stratified k‐fold cross‐validation.

The selected features were fed to an ordinary least squares (OLS) linear regression algorithm to model the relationship between brain connectivity and impulsivity. The predicted outcome (BIS‐11 score) for a given instance was determined as the weighted sum of its k features. The weights, represented by the beta coefficients that are learned for each feature represent each feature's influence on the predicted outcome. A stratified 10‐fold cross‐validation (with *K* = 10 folds) was conducted to evaluate the robustness of the trained model. While traditional cross‐validation methods suffice for classification tasks, they may inadequately address potential bias in the distribution of target variable values across folds in regression tasks, where the target variable is continuous. To ensure that each fold of the data contains a representative sample of the overall distribution of BIS‐11 scores, we used the quantile binning approach. This helps to ensure that the model is trained and evaluated on data that is similar in terms of the distribution of the relevant feature, leading to more accurate performance estimates. We used eight bins in this work, as presented in Figure 3, illustrating the bins and the number of subjects in each bin. With the target variable binned, we proceeded to perform stratified cross‐validation. The data was split into 10 folds using stratified sampling based on the target bins. A regression model was trained using data from nine folds. The model's performance was evaluated on the remaining fold. This procedure was repeated for all 10 folds, ensuring each fold was used for testing once. Finally, the performance scores across all 10 folds were averaged to obtain a more reliable estimate of the model's ability to generalize.

Subsequently, the feature importance for each fold was calculated, and finally, the final feature importance was estimated by averaging the feature importance scores from each fold. No regularization was applied because it may affect the interpretation and comparison of the feature importance scores (Ng 2004).

### 1.10 Model Evaluation

Several metrics were used to evaluate the model performance: mean absolute error (MAE), the root of mean squared error (RMSE), and *R* ^2^ value. MAE is calculated as the average of the absolute differences between actual and predicted values (Willmott and Matsuura 2005):

$$\mathrm{MAE}=\frac{1}{n}\sum_{i=1}^{n}|y_{i}-x_{i}|$$

RMSE is calculated as the average of the squares of the differences between actual and predicted values (Willmott and Matsuura 2005):

$$\text{RMSE}=\sqrt{\frac{1}{n}\sum_{i=1}^{n}{\left(y_{i}-x_{i}\right)}^{2}}$$

where *n* is the number of observations, *y*_*i*_ is the actual value for the *i*th observation; *x*_i_ is the predicted value for the *i*th observation.

*R* ^2^ is a measure of how well a regression model fits the data. It is calculated as the ratio of the explained variation to the total variation in the response variable (Kasuya 2019):

$$R^{2}=1-\frac{\mathrm{SSE}}{\mathrm{SST}}$$

SSE is the sum of squared errors, and SST is the sum of squares.

### 1.11 Feature Importance

Finally, we calculated the feature importance of each feature using the *t*‐statistic from the 10‐fold cross‐validated model to identify the most influential connectivity locations for impulsivity (Molnar 2022). The *t*‐statistic is the ratio of the estimated weight to its standard error (SE). It is given by:

$$t_{{\hat{\beta }}_{j}}=\frac{{\hat{\beta }}_{j}}{\mathrm{SE}\left({\hat{\beta }}_{j}\right)}$$

The importance of a feature ($t_{\hat{\beta }}$) increases with increasing weight ($\hat{\beta }$). The more variance the estimated weight has, the less certain we are about the correct value and the less important the feature is.

In addition, we assessed node degree (*k*), which is the total number of edges connected to a specific node. This metric was provided by the BioImage Suite Connectivity Viewer, which we used for visualization (see below).

### 1.12 Visualization and Network Characterization

Results were visualized using the BioImage Suite Web 1.2.0 software package (BioImage Suite Connectivity Viewer; bioimagesuiteweb.github.io). In line with the ROI‐to‐ROI functional connectivity analysis, nodes were defined by the Shen atlas (268 regions, Shen et al. 2013). Subsequently, the 268 nodes were categorized into 10 macroscale brain regions, as defined by the Shen atlas, including the prefrontal cortex (46 nodes), motor lobe (21 nodes), insular lobe (7 nodes), parietal lobe (27 nodes), temporal lobe (39 nodes), occipital lobe (25 nodes), limbic lobe (36 nodes), cerebellum (41 nodes), subcortical lobe (17 nodes), and brainstem (9 nodes). Finally, we computed the number of edges connecting each pair of macroscale regions.

## 2 Results

### 2.1 Normality Test

We first examined the distribution of BIS‐11 scores. According to a one‐sample Kolmogorov–Smirnov Test of normality, BIS‐11 scores were normally distributed (*D* = 0.08, *p* = 0.26; see Figure 1). BIS‐11 data ranged from 40 to 92 (*M* = 61.35, *SD* = 10.81) and were not significantly correlated with age (*r* = −0.04, *p* = 0.62).

### 2.2 Model Results

**FIGURE 4:**
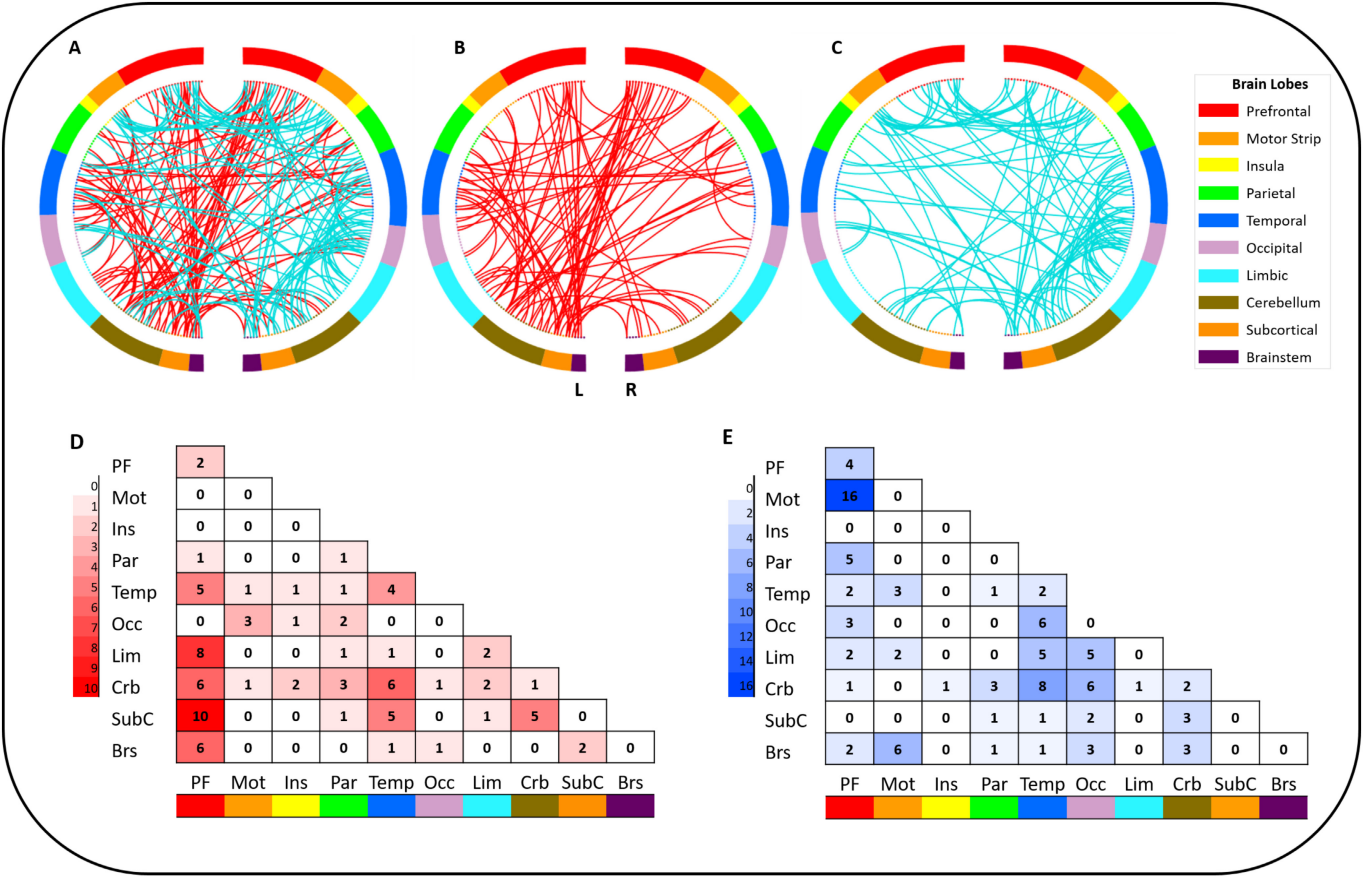
Functional connections predicting individual trait impulsivity. (A) all common edges contributing to the final model. (B) edges with a positive association with the BIS‐11. (C) edges with a negative association with the BIS‐11. (D and E) number of edges connecting each pair of macroscale brain regions contributing to the BIS‐11 network, for the positive (red) and negative (blue) associations, respectively.

**FIGURE 5:**
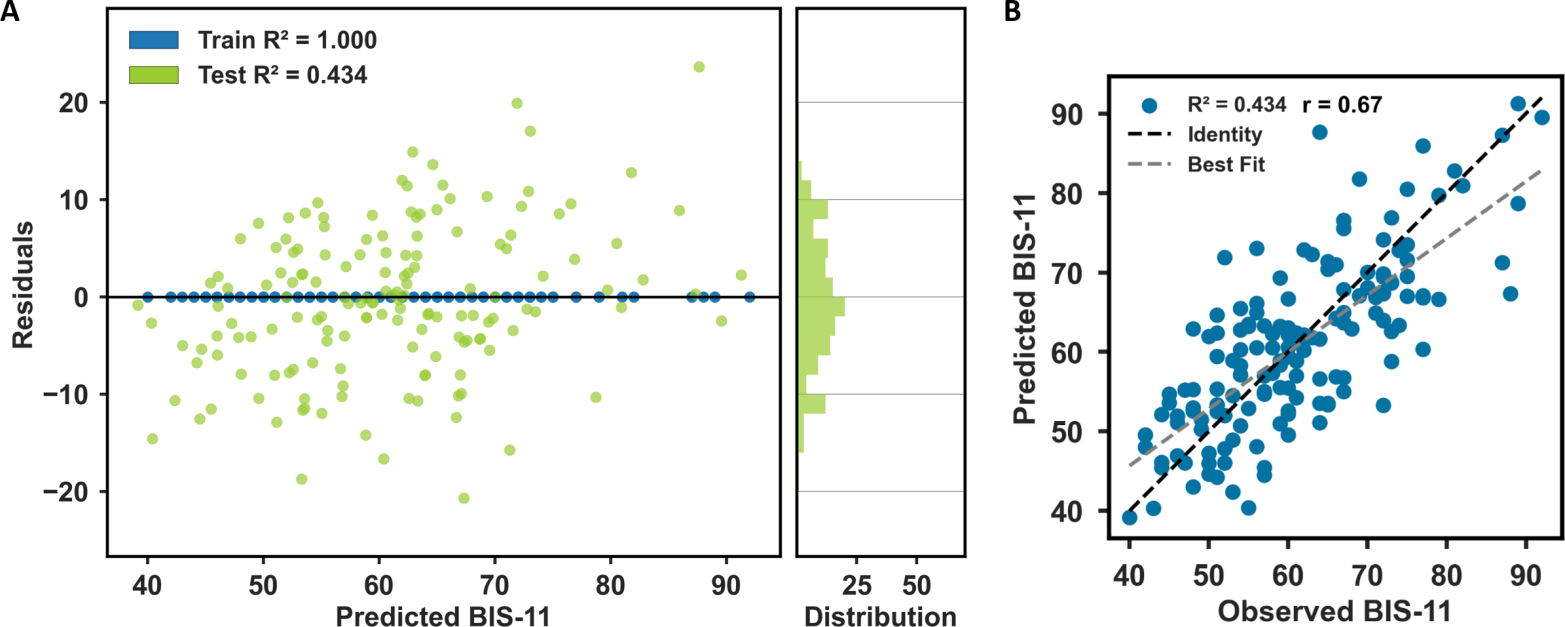
Model evaluation (A) predicted BIS‐11 scores and their residuals. (B) observed and predicted BIS‐11 scores.

Indices of the robustness of the trained model after 10‐fold cross‐validation may be found in Table 1. The final network containing edges that appeared in the top 60% of all edges across all repeats of repeated cross‐validation, contained 189 edges (Figure 4A). In total, 88 edges with a positive relationship and 101 edges with a negative relationship with the BIS‐11 contributed to the model. Connectivity between functional networks defined within the Shen atlas is presented separately for positive (Figure 4B) and negative correlations (Figure 4C). Furthermore, we investigated the number of edges between pairs of macroscale brain regions (separated for positive and negative BIS‐11 associations). Regions with the highest number of contributing edges included the prefrontal cortex with limbic, subcortical, cerebellar, and brainstem nodes as well as the prefrontal cortex with the motor strip (Figure 4D,E). Results of the model indicated that observed and predicted BIS‐scores correlated well with each other (*r* = 0.67, *R* ^2^ = 0.43; see Figure 5).

**TABLE 1:** Model evaluation matrices after 10‐fold cross‐validation.

	MAE	RMSE	*R* ^2^
Mean	6.048	7.562	0.434
SD	0.729	0.775	0.169

*Note:* Matrices are averaged across the results of the 10‐folds.

Abbreviations: MAE = mean absolute error, MAPE = mean absolute percentage error, MSE = mean square error, RMSLE = root mean square error, SD = standard deviation.

**FIGURE 6:**
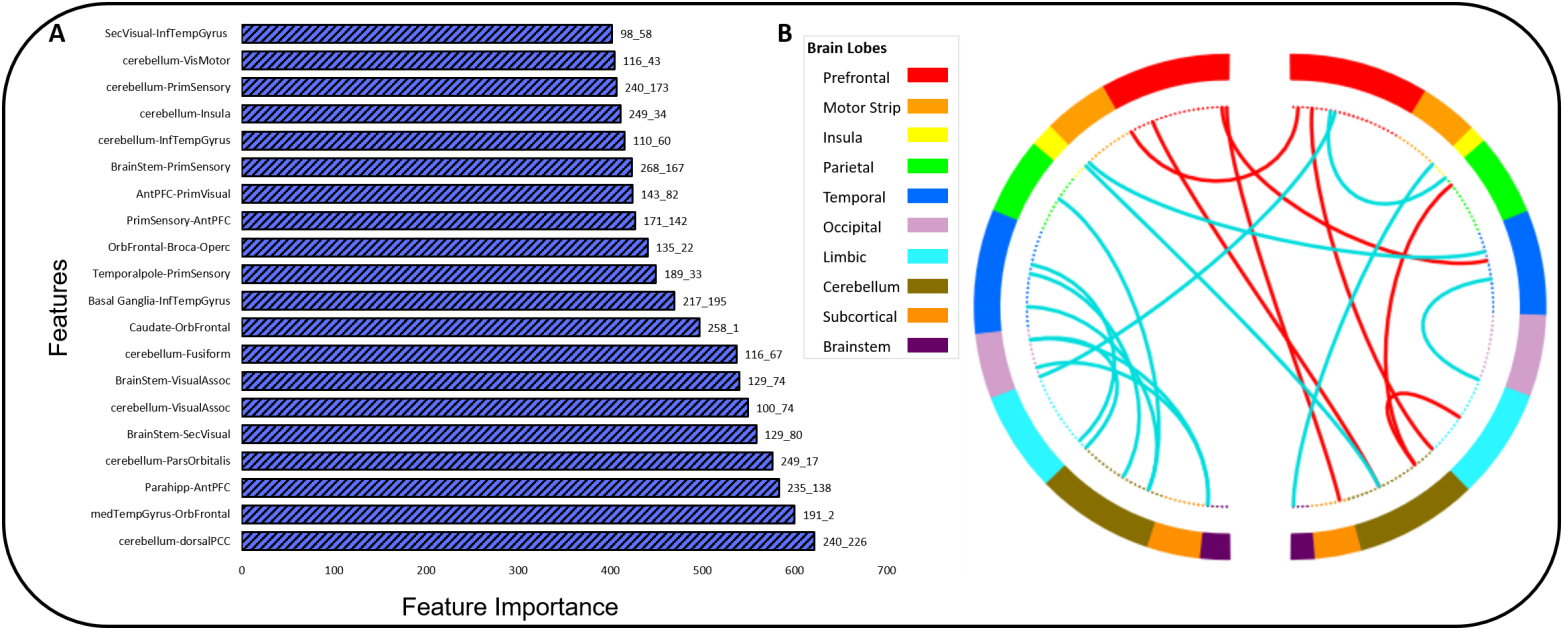
Top 20 node pairs of the model as determined by their feature importance (A). (B) Macroscale brain regions of most important features. Red lines indicate positive BIS‐11 associations and turquoise lines indicate negative BIS‐11 associations.

The top 20 features with the greatest feature importance as assessed by the *t*‐statistic included edges located in the cerebellum, the prefrontal cortex, the occipital cortex, and limbic areas (see Table 2 for more fine‐grained neuroanatomical descriptions and Figure 6 for visualization). These edges were also among the 15 most highly connected nodes in the network as assessed by node degree (see Table S1 for the top 15). The most highly connected node, as determined by node degree, was node 116 (*k* = 8).

**TABLE 2:** Top 20 features of the final model.

Pair of nodes	Region	Hemi	Network name	MNI	Association	Node degree of individual node
240	Cerebellum	l	cerebellum	−21.24, −70.02, −48.88		**6**
226	Limbic	l	dorsalPCC	−8.79, −42.55, 50.11	pos	1
191	Temporal	l	medTempGyrus	−58.98, −29.96, 3.49		2
2	Prefrontal	r	OrbFrontal	9.57, 17.75, −19.5	pos	**7**
235	Limbic	l	Parahipp	−21.38, −4.06, −29.37		**6**
138	Prefrontal	l	AntPFC	−6.93, 48.31, −5.71	pos	1
249	Cerebellum	l	cerebellum	−34.64, −50.33, −53.98		3
17	Prefrontal	r	ParsOrbitalis	33.4, 37.28, −16.43	pos	1
129	Brainstem	r	BrainStem	4.75, −37.16, −53.01		**5**
80	Occipital	r	SecVisual	7.83, −88.59, 11.86	neg	4
100	Cerebellum	r	cerebellum	32.22, −78.45, −40.43		2
74	Occipital	r	VisualAssoc	45.08, −74.29, 2.58	neg	2
129	Brainstem	r	BrainStem	4.75, −37.16, −53.01		**5**
74	Occipital	r	VisualAssoc	45.08, −74.29, 2.58	neg	2
116	Cerebellum	r	cerebellum	41.9, −63.98, −49.17		**8**
67	Temporal	r	Fusiform	36.45, −69.08, −17.46	neg	3
258	Subcortical	l	Caudate	12.52, 11.62, 8.68		**6**
1	Prefrontal	r	OrbFrontal	13.86, 56.85, −16.64	pos	4
217	Limbic	l	Basal Ganglia	−23.59, −41.29, 19.92		2
195	Temporal	l	InfTempGyrus	−37.84, −13.23, −29.26	neg	4
189	Temporal	l	Temporalpole	−22.92, 9, −38.79		1
33	Motostrip	r	PrimSensory	41.97, −23.38, 53.41	neg	2
135	Prefrontal	l	OrbFrontal	−18.22, 19.05, −20.98		3
22	Prefrontal	r	Broca‐Operc	39.98, 17.61, 29.19	pos	2
171	Parietal	l	PrimSensory	−50.64, −23.79, 41.37		2
142	Prefrontal	l	AntPFC	−29.21, 54.27, 2.51	neg	2
143	Prefrontal	l	AntPFC	−42.72, 47.25, −6.93		2
82	Occipital	r	PrimVisual	14.64, −68.33, 8.28	neg	4
268	Brainstem	l	BrainStem	−6.12, −18.93, −36.77		4
167	Motorstrip	l	PrimSensory	−35.88, −23.3, 65.6	neg	3
110	Cerebellum	r	cerebellum	−21.07, −54.82, −23.76		2
60	Temporal	r	InfTempGyrus	31.46, 0.67, −44.44	neg	3
249	Cerebellum	l	cerebellum	−34.64, −50.33, −53.98		3
34	Insula	r	Insula	41.79, 4.97, −7.62	neg	1
240	Cerebellum	l	cerebellum	−21.24, −70.02, −48.88		**6**
173	Parietal	l	PrimSensory	−41.23, −15.57, 14.45	pos	1
116	Cerebellum	r	cerebellum	41.9, −63.98, −49.17		**8**
43	Parietal	r	VisMotor	31.62, −60.79, 49.21	neg	4
98	Limbic	r	SecVisual	14.6, −46.08, 2.84		1
58	Temporal	r	InfTempGyrus	40.31, −11.27, −35.81	neg	2

*Note:* Top 20 node pairs in the model as determined by their feature importance (*t*‐statistic). Node degree in bold are among the top 15 nodes with the greatest connections. Node 116 is the most highly connected node as determined by node degree (*k* = 8).

Abbreviations: Hemi = hemisphere, l = left, r = right, MNI = Montreal Neurological Institute coordinates.

## 3 Discussion

In this study, we employed a data‐driven approach to construct a neural network of trait impulsivity by analyzing whole‐brain functional connectomes. The employed machine learning algorithm excels at identifying complex relationships and patterns within data. Our findings reveal a widespread neural network associated with BIS‐11 scores and a notable correlation between predicted and observed scores (*r* = 0.67, *R* ^2^ = 0.434), aligning with the higher end of the range of correlations typically reported (between 0.2 and 0.5) in prior studies employing similar linear, predictive connectome‐based modeling approaches to elucidate brain‐behavior relationships (Shen et al. 2017).

Importantly, our predictive model encompasses both positive and negative associations with BIS‐11 scores. Specifically, connectivity strength between the prefrontal cortex and subcortical structures/the limbic system, the cerebellum, and brainstem regions was positively associated with impulsivity scores. Within the entire impulsivity network of the positive associations, especially the orbitofrontal cortex, and anterior prefrontal cortex (part of the prefrontal lobe), the parahippocampus (limbic), the striatum (subcortical) and the cerebellum were central nodes as indexed by their feature importance and node degree. Regarding negative associations, especially connections between the prefrontal cortex and the motor strip (i.e., premotor/supplementary motor area) as well as connections including the cerebellum and the temporal lobe emerged. Feature importance and node degree emphasize the significance of the cerebellum and brainstem as pivotal nodes in this context.

Building upon prior research, our identified nodes and edges can be categorized into networks widely recognized for their involvement in impulsivity, as well as networks that have thus far received less attention in impulsivity research.

Specifically, connections that exhibited a positive association with BIS‐11 scores can be attributed to the basal ganglia‐thalamo‐prefrontal circuit. These brain regions work together to regulate cognitive function and motor control, which are essential for inhibiting impulsive behavior (Bari and Robbins 2013; Dalley and Robbins 2017). The involvement of the basal ganglia‐thalamo‐prefrontal circuit in impulsivity is further supported by recent evidence from animal studies (Guzulaitis and Palmer 2023). In humans, lower prefrontal grey matter volume and greater resting‐state functional connectivity within this network have been previously associated with greater trait impulsivity in healthy adults (Korponay et al. 2017). Of this network, the striatum and the orbitofrontal cortex, hold significance for reward‐related aspects of impulsivity including risk‐taking behavior and reward impulsivity as evidenced by fMRI (Angelides, Gupta, and Vickery 2017; Misonou and Jimura 2021; Rolls et al. 2022) and structural brain studies (Cho et al. 2013; Wang et al. 2020). Furthermore, recent meta‐analytic evidence has highlighted the functional connections between the dorsal striatum and the prefrontal cortex, which are involved in several different facets of impulsivity, as demonstrated through conjunction analysis (Mattavelli et al. 2024). The relevance of the striatum and the orbitofrontal cortex for trait impulsivity is underscored by both node degree and feature importance in our study. Recently, a graph‐theoretical predictive model has emphasized the centrality (a graph theory index for the importance of a region) of the anterior cingulate cortex in predicting impulsivity (Dan et al. 2024). Similarly, in our study, the anterior prefrontal cortex ranked among the top 20 most important features. Together, these findings corroborate and further substantiate the existing evidence indicating heightened functional connectivity within the basal ganglia‐thalamo‐prefrontal network in relation to impulsivity.

Edges that showed negative associations with the BIS‐11 included connections between the prefrontal cortex and the motor strip. The prefrontal cortex is recognized for its vital role in regulating impulsive behaviors via its connections with the motor strip (Borgomaneri, Serio, and Battaglia 2020; Duann et al. 2009; Rae et al. 2015). Especially the role of the pre‐supplementary motor area (pre‐SMA) in response inhibition has been extensively acknowledged, particularly in conjunction with a neural network that encompasses the prefrontal cortex, striatal, and subthalamic regions (Aron 2011; Dambacher et al. 2014). Extending the traditional view that the pre‐SMA is primarily responsible for inhibitory aspects of response control, more recent evidence suggests that, in concert with the prefrontal cortex, the pre‐SMA may rather represent a motivational signal for specific actions but does not control whether these actions are executed (Lohse et al. 2023; Scangos and Stuphorn 2010; Whelan et al. 2012). Interestingly, a study on risk‐taking behavior demonstrated that the pre‐SMA is centrally involved in translating trait impulsivity into actual impulsive behavior (Lohse et al. 2023). The authors reported an increase in risk‐taking behavior in low impulsive individuals after repetitive transcranial magnetic stimulation of the pre‐SMA. This effect was reversed in high impulsive individuals and echoed in rTMS‐induced changes in pre‐SMA activity. Notably, most evidence regarding prefrontal‐motor strip associations comes from research on inhibitory control, while our resting‐state connectivity findings suggest a more general importance of this network for impulsivity.

In addition to the regions commonly associated with impulsivity, our analysis underscores the importance of nodes within the cerebellum and brainstem, whose connections demonstrate both positive and negative associations with the BIS‐11. Historically, the cerebellum has been associated with its role in the vestibulomotor system (Timmann et al. 2010). However, recent research highlights its contributions to higher cognitive functions and emotion processing (Adamaszek et al. 2017; Schmahmann 2019; Timmann et al. 2010). Neuroanatomical studies reveal extensive connections between the cerebellum and the prefrontal cortex via thalamic nuclei, indicating its involvement beyond motor control (Manto et al. 2012; Middleton and Strick 2001). The results of our analyses suggest that the cerebellum serves as a core node within several networks and is involved in connections that exhibit both positive and negative associations with self‐reported impulsivity. It emerged as a high‐degree node and was among the 20 most important features corroborating recent literature acknowledging the cerebellum's role in impulsivity (Abdallah et al. 2020; Miquel et al. 2019). On the one hand, subcortical‐cerebellar connectivity seems to be positively associated with self‐reported impulsivity. This finding is in line with previous research suggesting increased connectivity between the striatum and cerebellum may be linked to reduced avoidance of actions with potential negative consequences (Abdallah et al. 2020). On the other hand, we also identified nodes within the cerebellum exhibiting negative BIS‐11 associations. Specifically, and rather unexpected, connections between the cerebellum and the temporal as well as the occipital lobe emerged. Occipital and temporal nodes involved in these edges mainly comprised brain areas implicated in visual networks (e.g., medial temporal gyrus, inferior temporal gyrus, fusiform gyrus, visual association areas). Some of these cerebellar connections have been mentioned in the context of concepts related to impulsivity. In this vein, temporal‐cerebellar connections, for example, have been shown to be involved in action‐feedback monitoring (van Kemenade et al. 2019). Although these connections were unexpected, this result highlights human behavior is complex and typically arises from communication across multiple networks, rather than solely within or between individual pairs of brain regions (Liégeois et al. 2019).

Our findings suggest that impulsivity is a multifaceted trait shaped by multiple brain networks. Notably, we observed a greater number of edges with a negative relationship to impulsivity than those with a positive relationship. This predominance of negative BIS associations could imply that a larger proportion of connections within the impulsivity network are involved in inhibitory control mechanisms. The prevalence of negative associations may reflect the brain's attempt to regulate or suppress impulsive behaviors. Alternatively, this could indicate that the neural circuits associated with higher impulsivity—those positively correlated with impulsivity—are less robust and, therefore, less prominent in our overall impulsivity network.

As a transdiagnostic symptom, dysfunctional impulsivity poses a substantial burden on patients with mental disorders and the health system. While our results, which are based on healthy individuals, may not directly extend to individuals with mental disorders, they may provide a reference into the neural mechanisms of pathological impulsivity. In fact, some of the discussed networks are known to show alteration in impulsivity‐related disorders. For example, reduced functional connectivity between the prefrontal cortex and motor‐strip areas is observed in impulsivity‐related disorders such as Internet Gaming Disorder (Dong et al. 2021) and ADHD (Kim et al. 2023; Sotnikova et al. 2017). Additionally, alterations in cerebellar metabolism, structure, and function are implicated in various mental disorders characterized by heightened impulsivity, including ADHD, autism spectrum disorder or borderline personality disorder (De Vidovich et al. 2016; Hendriksen & Vles 2008; King et al. 2003; Lupo et al. 2018; Pierce and Courchesne 2001). Furthermore, impulsivity exacerbates treatment outcomes and increases the likelihood of substance abuse initiation and relapse (Rosvall et al. 2008; Stevens et al. 2015). After all, a comprehensive understanding of functional brain circuits associated with high and/or dysfunctional impulsivity may pave the way toward more effective prevention and intervention techniques. Assessing feature importance and node degree can particularly inform the development of interventions targeting identified network hubs that may hold specific clinical relevance. For instance, non‐invasive neuromodulation techniques such as neurofeedback, transcranial direct current stimulation and transcranial magnetic stimulation could specifically target identified nodes or edges.

### 3.1 Limitations and Future Directions

Whilst holding important implications, our study faces some limitations. Impulsivity is a multi‐dimensional construct, and it is plausible that distinct (and overlapping) brain networks contribute to different aspects of impulsivity. For example, a large study on adolescents identified distinct neural endophenotypes and genetic factors to be related to different aspects of impulsive behavior (Whelan et al. 2012). Thus, characterizing individuals solely by the BIS‐11 questionnaire might oversimplify the complexity of impulsivity. Moreover, the assumption of linearity in the relationship between brain connectivity and impulsivity may oversimplify the complex nature. Furthermore, since our sample includes only males (two of the contributing projects only included males for their specific research question), interpretations of results are limited to men and may not be generalized to women. Given the gender gap in medical and neuroscience research (Merone et al. 2022; Zugman et al. 2023), further research to characterize the brain‐behavior relationship of impulsivity in females is needed. Interestingly, recent findings demonstrated significant gender differences in fMRI resting‐state networks of impulsive decision‐making (Lv et al. 2023).

Moreover, it is warranted that our model may be validated in a new independent sample to investigate its robustness and extended to clinical samples. Overall, additional validation, robustness checks, and alternative modeling approaches are needed to mitigate these limitations and ensure the reliability and validity of the findings.

## 4 Conclusion

By using a whole‐brain approach, we were able to reveal an impulsivity model characterized by a large number of connections across diverse networks, highlighting its complexity. More specifically, results of our model support previous findings implicating the basal ganglia‐thalamo‐prefrontal network in impulsivity. Furthermore, we extended this evidence by emphasizing the significance of brain areas within the impulsivity network that have previously been overlooked, notably the cerebellum, brainstem, and the temporal lobe. This sheds light on their importance as key nodes. By elucidating the intricate brain‐behavior relationship of functional impulsivity dimensions, we lay the groundwork for uncovering alterations in functional brain networks associated with dysfunctional impulsivity.

## Ethics Statement

Ethics approval has been obtained for all three studies contributing to the data analyzed in the current manuscript.

## Conflicts of Interest

The authors declare no conflicts of interest.

## Supporting information


**TABLE S1.** Node Degree Top 15.

## Data Availability

The data that support the findings of this study are available from the corresponding author upon reasonable request.
