# Supplementary material for: Functional Brain Network of Trait Impulsivity: Whole‐Brain Functional Connectivity Predicts Self‐Reported Impulsivity
Source: Hum Brain Mapp. 2024 Oct 29;45(15):e70059. doi: 10.1002/hbm.70059 (PMC11519747; doi:10.1002/hbm.70059)
Supplement: Supplementary file 1 — TABLE S1. Node Degree Top 15. [file HBM-45-e70059-s001.docx]

Supplementary Material

Table S1 Node Degree Top 15

| **#** | **Node** | **Details** |
| --- | --- | --- |
| 1 | 116 | Degree=8 (MNI = 41.9, -63.98, -49.17), R Cerebellum |
| 2 | 2 | Degree=7 (MNI = 9.57, 17.75, -19.5), R Prefrontal |
| 3 | 258 | Degree=6 (MNI = -12.52, 11.62, 8.68), L Subcortical |
| 4 | 240 | Degree=6 (MNI = -21.24, -70.02, -48.88), L Cerebellum |
| 5 | 235 | Degree=6 (MNI = -21.38, -4.06, -29.37), L Limbic |
| 6 | 185 | Degree=6 (MNI = -38.01, 6.07, -37.86), L Temporal |
| 7 | 166 | Degree=6 (MNI = -27.58, -9.08, 55.86), L MotorStrip |
| 8 | 8 | Degree=6 (MNI = 44.56, 46.19,-4.9), R Prefrontal |
| 9 | 262 | Degree=5 (MNI = -9.59, -25.43,-1.42), L Subcortical |
| 10 | 148 | Degree=5 (MNI = -11.17, 34.26, 51.58) L Prefrontal |
| 11 | 140 | Degree=5 (MNI = -5.96, 48.09, 11.72) L Prefrontal |
| 12 | 137 | Degree=5 (MNI = -8.15, 39.69, -21.44) L Prefrontal |
| 13 | 130 | Degree=5 (MNI = 9.67, -18.71, -30.66) R Brainstem |
| 14 | 129 | Degree=5 (MNI = 4.75, -37.16,-53.01) R Brainstem |
| 15 | 93 | Degree=5 (MNI = 28.82,-36.92,-0.03) R Limbic |

*Note.* MNI = Montreal Neurological Institute, R = right, L = left
